# Supplementary material for: Evaluation of SARS-CoV-2 in semen, seminal plasma, and spermatozoa pellet of COVID-19 patients in the acute stage of infection
Source: PLoS One. 2021 Dec 14;16(12):e0260187. doi: 10.1371/journal.pone.0260187 (PMC8670700; doi:10.1371/journal.pone.0260187)
Supplement: S1 Table — (DOCX) [file pone.0260187.s001.docx]

**S1 Table: Clinical characteristics of the 32 enrolled COVID-19 patients.**

| **Patient** | **Age**  **(years old)** | **BMI^a^**  **(kg/m^2^)** | **Smoking** | **Chronic pathologies** | **Nature of symptoms** | **Specific COVID treatment** | **Place of semen collection** |
| --- | --- | --- | --- | --- | --- | --- | --- |
| 1 | 28 | 21.4 | Never | None | Dyspnea, cough, expectoration, diarrhea | None | Home |
| 2 | 58 | 22.4 | Never | None | Fever (38°C), myalgia | None | Home |
| 3 | 40 | 27.5 | Never | None | Cough, expectoration, headache, myalgia, nausea, diarrhea, anosmia, fever (38°C) | None | Hospital |
| 4 | 21 | 21.8 | Current | None | Cough | None | Hospital |
| 5 | 35 | 30.1 | Former | None | Headache, anosmia | Analgesics | Hospital |
| 6 | 47 | 34.1 | Never | None | Asymptomatic (contact case) | None | Hospital |
| 7 | 33 | 24.2 | Never | None | Myalgia, anosmia, ageusia | None | Hospital |
| 8 | 54 | 30.4 | Never | Arterial hypertension | Cough, chest pain, fever (39°C), myalgia, diarrhea | Analgesics | Hospital |
| 9 | 30 | 26.2 | Current | None | Cough, expectoration, fever (38°C), headache, anosmia | Analgesics | Hospital |
| 10 | 51 | 27.2 | Never | None | Cough, fever (38.7°C), headache | Analgesics | Hospital |
| 11 | 54 | 24.1 | Never | Vasectomy | Dyspnea, chest pain, headache, myalgia, fever (38°C) | None | Hospital |
| 12 | 31 | 25.7 | Never | None | Cough, headache | None | Hospital |
| 13 | 58 | 30.0 | Never | HIV (antiretroviral) | Dyspnea, cough, chest pain, fever, headache, myalgia | Antibiotics, vitamin D | Hospital |
| 14 | 39 | 25.2 | Never | None | Fever, dyspnea, cough, chest pain, headache, myalgia, diarrhea, anosmia, ageusia | Analgesics | Hospital |
| 15 | 41 | 23.4 | Never | None | Asymptomatic (contact case) | None | Hospital |
| 16 | 21 | 21.9 | Never | Vision disorders | Asymptomatic (contact case) | None | Hospital |
| 17 | 40 | 26.9 | Never | None | Fever, headache, myalgia | Analgesics | Hospital |
| 18 | 25 | 23.7 | Former | Chronic respiratory disease | Cough, headache, myalgia, nausea, diarrhea | Analgesics | Hospital |
| 19 | 39 | 32.0 | Never | None | Asymptomatic (contact case) | None | Hospital |
| 20 | 43 | 27.2 | Former | None | Headache, myalgia | Analgesics | Hospital |
| 21 | 42 | 29.3 | Current | None | Fever, headache, myalgia, diarrhea, nausea | None | Hospital |
| 22 | 52 | 39.8 | Never | Arterial hypertension | Asymptomatic (contact case) | None | Home |
| 23 | 28 | 22.6 | Never | None | Cough, expectoration, chest pain, headache | None | Hospital |
| 24 | 47 | 28.1 | Never | None | Dyspnea, cough, headache, myalgia | None | Hospital |
| 25 | 48 | 30.4 | Current | Anxiety | Dyspnea, expectoration, chest pain, headache, myalgia | Analgesics | Hospital |
| 26 | 34 | 21.6 | Never | None | Cough, diarrhea | None | Hospital |
| 27 | 25 | 20.5 | Never | None | Anosmia, ageusia | Analgesics | Hospital |
| 28 | 34 | 26.1 | Current | Type 2 diabetes | Asymptomatic (contact case) | None | Hospital |
| 29 | 21 | 23.7 | Current | None | Cough, headache, myalgia, nausea, diarrhea, fever (38.7°C) | Analgesics | Hospital |
| 30 | 36 | 28.1 | Never | None | Cough, chest pain, headache, myalgia | None | Hospital |
| 31 | 40 | 24.5 | Never | Chronic respiratory disease | Fever (38°C), myalgia | None | Hospital |
| 32 | 47 | 32.4 | Never | Diabetes | Cough, nausea | None | Hospital |

*^a^ BMI: body mass index*

This table gives the medical characteristics of the 32 enrolled COVID-19 patients including demographic data, chronic pathologies, symptoms, specific COVID-19 treatments and place of semen collection.
