# Supplementary material for: Evaluation of SARS-CoV-2 in semen, seminal plasma, and spermatozoa pellet of COVID-19 patients in the acute stage of infection
Source: PLoS One. 2021 Dec 14;16(12):e0260187. doi: 10.1371/journal.pone.0260187 (PMC8670700; doi:10.1371/journal.pone.0260187)
Supplement: Attachment — Submitted filename: 2021-10-01 Responses to reviewers.docx [file pone.0260187.s003.docx]

**RESPONSE TO THE REVIEWERS**

Reviewers' comments:

Reviewer's Responses to Questions

**Comments to the Author**

1. Is the manuscript technically sound, and do the data support the conclusions ?

The manuscript must describe a technically sound piece of scientific research with data that supports the conclusions. Experiments must have been conducted rigorously, with appropriate controls, replication, and sample sizes. The conclusions must be drawn appropriately based on the data presented.

Reviewer #1: Yes

Reviewer #2: Yes

Reviewer #3: Yes

2. Has the statistical analysis been performed appropriately and rigorously?

Reviewer #1: Yes

Reviewer #2: N/A

Reviewer #3: Yes

3. Have the authors made all data underlying the findings in their manuscript fully available?

The [PLOS Data policy](http://www.plosone.org/static/policies.action#sharing) requires authors to make all data underlying the findings described in their manuscript fully available without restriction, with rare exception (please refer to the Data Availability Statement in the manuscript PDF file). The data should be provided as part of the manuscript or its supporting information, or deposited to a public repository. For example, in addition to summary statistics, the data points behind means, medians and variance measures should be available. If there are restrictions on publicly sharing data—e.g. participant privacy or use of data from a third party—those must be specified.

Reviewer #1: Yes

Reviewer #2: Yes

Reviewer #3: Yes

4. Is the manuscript presented in an intelligible fashion and written in standard English?

PLOS ONE does not copyedit accepted manuscripts, so the language in submitted articles must be clear, correct, and unambiguous. Any typographical or grammatical errors should be corrected at revision, so please note any specific errors here.

Reviewer #1: No

Reviewer #2: Yes

Reviewer #3: Yes

5. Review Comments to the Author

Please use the space provided to explain your answers to the questions above. You may also include additional comments for the author, including concerns about dual publication, research ethics, or publication ethics. (Please upload your review as an attachment if it exceeds 20,000 characters)

**Reviewer #1: Reviewer’s report**

Title: Evaluation of SARS-CoV-2 in semen, seminal plasma, and final fraction with spermatozoa of COVID-19 patients at acute stage of infection
Date: 31 August 2021
Manuscript ID: PONE-D-21-24028
Reviewer’s report:
In the manuscript titled “: Evaluation of SARS-CoV-2 in semen, seminal plasma, and final fraction with spermatozoa of COVID-19 patients at acute stage of infection” the authors evaluated the presence of SARS-CoV-2 in semen, seminal plasma and pallet samples of COVID-19 positive patients in the acute phase of infection. The topic could be of interest however several concerns need to be raised:

1- I suggest changing the “native semen” to semen which is more common.
Ok we have changed the denomination as suggested.

2- Do the authors mean pallet after spinning the semen sample by “final fraction containing spermatozoa”? If yes change it to pallet. Ok we have changed the denomination « final fraction containing spermatozoa » by « spermatozoa pellet » as suggested.

3- Please write down these expressions: ESHRE, IFFS, ASRM in the instruction. As we have shortened the introduction, these abbreviations no longer appear. It is therefore no longer necessary to detail them.

4- Introduction is too long. I recommend shortening it. We have deleted the second last paragraph which was not useful for the presentation of the context of the study.

5- Please clarify and explain which criteria did the authors considered as an acute phase and moderate symptom? The acute phase is already described in the introduction (line 88 - ≤ 8 days after the onset of symptoms) but we added its definition in materials and methods as well as the definition of moderate symptoms. See lines 261-264.

6- Discussion lacks depth (it is purely descriptive). We added a paragraph to further discussion. See lines 217-228 in yellow.

7- It is noted that this manuscript needs a professional technical English editing service paying particular attention to English grammar and sentence structure. On the 4th of august, Plos one suggested us to copyedit our manuscript for language usage, spelling, and grammar, what we did by our translation provider ACOLAD. We submitted the english-revised manuscript to Plos one on the 14th august and their internal editors approved it on the 17th of august.

**Reviewer #2:** The article from L. Delaroche et al. addresses the question of the presence of SARS-Cov-2 in semen from COVID-19 patients in the acute stage of infection. This study is an observational study.
Thirty-two men were included in the study and the virus was detected by RT-qPCR using the RealStar RT-PCR kit in native semen, seminal plasma and 80% fraction obtained after centrifugation of the semen on discontinuous density gradients. Only one volunteer presented positive SARS-Cov-2 RT-PCR in semen and seminal plasma, 80%fraction were negative. Following bacterial investigation of semen samples, the authors suggested that the SARS-Cov-2 presence in semen from one volunteer is more a consequence of oral contamination than e semen SARS-Cov-2 infection.

I have several comments:

I did not understand the reasons of semen home collection in 3 patients. The authors explain this: “according to the patient’s clinical condition”. This point needs to be clarified as 2 patients were symptomatic and the last was asymptomatic. Home collection was preferred for patients who had difficulties in collecting semen in the laboratory. We clarified this point in line 257.

The semen investigation methods are not totally comprehensives. Linea 261: is the supernatant obtained from native semen centrifugation or from density gradient centrifugation? If is the first case, I suggest to present before “the remaining semen samples…(linea 258). Before preparing semen, we kept an aliquot of 200 µl native semen sample. Then, the remaining semen sample was prepared by a two-steps discontinuous density-gradient to separate the seminal plasma and the spermatozoa pellet. We modified the paragraph as suggested. See in lines 271-272.

Table 1 and 2 reported the result characteristics in detail. For the reader, I think that table 1 could be more synthetic. We agree that the Table 1 was too complex, and moved it as a supplementary material (now Supplementary Table 1).

One patient had a positive semen. The patient performed semen probe at home and the precise bacterial study suggest a possible contamination from oropharyngeal secretions. In discussion, the authors mentioned the “The bacteria found can neither confirm nor strictly rule out a weak contamination of the semen sample by oropharyngeal secretions”. Indeed, higher concentrations of bacterial DNA were found in this specimen than in others but we did not know the levels which allows to conclude oral contamination in one and no in others. The sentence in abstract “the bacteria identified do not clearly rule out contamination by oropharyngeal secretions” could be modified according to “neither confirm nor rule out contamination” according to discussion. We modified the sentence as suggested. See in Line 33.

**Reviewer #3**: A study with a final sample size of 32 COVID-19 positive volunteers aimed to determine whether SARS-CoV-2 could be detected in native semen, seminal plasma, and final fraction with spermatozoa samples. SARS-CoV-2 was detected in only one volunteer’s native semen and seminal plasma samples. The results are primarily descriptive.

Minor revisions:
1- Line 104: Indicate the type of summary statistics provided for age. This is the average age. We changed the sentence as suggested. See in Line 106.

2- Patient characteristics: In addition to the frequencies, state the corresponding percentages. We added the percentages as suggested. See lines 106-115.

3- Line 186: Provide the corresponding percentage and a 95% confidence interval for the 31/32 result. We added the percentage as suggested. See line 189. However, it is not possible to calculate the 95th confidence interval.

6. PLOS authors have the option to publish the peer review history of their article. If published, this will include your full peer review and any attached files.


If you choose “no”, your identity will remain anonymous but your review may still be made public.

**Do you want your identity to be public for this peer review?** For information about this choice, including consent withdrawal, please see our [Privacy Policy](https://www.plos.org/privacy-policy).

Reviewer #1: **Yes:** kajal Khodamoradi

Reviewer #2: **Yes:** Bujan Louis

Reviewer #3: No
